# A 46,XY Female DSD Patient with Bilateral Gonadoblastoma, a Novel SRY Missense Mutation Combined with a WT1 KTS Splice-Site Mutation

**DOI:** 10.1371/journal.pone.0040858

**Published:** 2012-07-18

**Authors:** Remko Hersmus, Yvonne G. van der Zwan, Hans Stoop, Pascal Bernard, Rajini Sreenivasan, J. Wolter Oosterhuis, Hennie T. Brüggenwirth, Suzan de Boer, Stefan White, Katja P. Wolffenbuttel, Marielle Alders, Kenneth McElreavy, Stenvert L. S. Drop, Vincent R. Harley, Leendert H. J. Looijenga

**Affiliations:** 1 Department of Pathology, Erasmus MC - University Medical Center Rotterdam, Josephine Nefkens Institute, Daniel den Hoed Cancer Center, Rotterdam, The Netherlands; 2 Molecular Genetics and Development Division, Prince Henry’s Institute of Medical Research, Clayton, Victoria, Australia; 3 Department of Anatomy and Cell Biology, The University of Melbourne, Victoria, Australia; 4 Department of Clinical Genetics, Erasmus MC - University Medical Center Rotterdam, Rotterdam, The Netherlands; 5 Centre for Reproduction and Development, Monash Institute of Medical Research, Clayton, Victoria, Australia; 6 Department of Pediatric Urology, Erasmus MC - University Medical Center Rotterdam, Rotterdam, The Netherlands; 7 Department of Clinical Genetics, Academic Medical Center, University of Amsterdam, Amsterdam, The Netherlands; 8 Human Developmental Genetics Unit, Institute Pasteur, Paris, France; 9 Department of Pediatric Endocrinology, Erasmus MC - University Medical Center Rotterdam, Sophia Children’s Hospital, Rotterdam, The Netherlands; University of Bristol, United Kingdom

## Abstract

Patients with Disorders of Sex Development (DSD), especially those with gonadal dysgenesis and hypovirilization are at risk of developing malignant type II germ cell tumors/cancer (GCC) (seminoma/dysgerminoma and nonseminoma), with either carcinoma *in situ* (CIS) or gonadoblastoma (GB) as precursor lesion. In 10–15% of 46,XY gonadal dysgenesis cases (i.e., Swyer syndrome), *SRY* mutations, residing in the HMG (High Mobility Group) domain, are found to affect nuclear transport or binding to and bending of DNA. Frasier syndrome (FS) is characterized by gonadal dysgenesis with a high risk for development of GB as well as chronic renal failure in early adulthood, and is known to arise from a splice site mutation in intron 9 of the Wilms’ tumor 1 gene (*WT1*). Mutations in *SRY* as well as *WT1* can lead to diminished expression and function of SRY, resulting in sub-optimal *SOX9* expression, Sertoli cell formation and subsequent lack of proper testicular development. Embryonic germ cells residing in this unfavourable micro-environment have an increased risk for malignant transformation. Here a unique case of a phenotypically normal female (age 22 years) is reported, presenting with primary amenorrhoea, later diagnosed as hypergonadotropic hypogonadism on the basis of 46,XY gonadal dygenesis with a novel missense mutation in *SRY*. Functional *in vitro* studies showed no convincing protein malfunctioning. Laparoscopic examination revealed streak ovaries and a normal, but small, uterus. Pathological examination demonstrated bilateral GB and dysgerminoma, confirmed by immunohistochemistry. Occurrence of a delayed progressive kidney failure (focal segmental glomerular sclerosis) triggered analysis of *WT1*, revealing a pathogenic splice–site mutation in intron 9. Analysis of the *SRY* gene in an additional five FS cases did not reveal any mutations. The case presented shows the importance of multi-gene based diagnosis of DSD patients, allowing early diagnosis and treatment, thus preventing putative development of an invasive cancer.

## Introduction

Disorders of Sex development (DSD) are congenital conditions of incomplete or disordered gonadal development leading to discordance between genetic sex, gonadal sex, and phenotypic sex [1]. DSD occurs with an estimated incidence of 1∶5000 [1]. Individuals with an underlying DSD, especially those with specific Y chromosomal material in their karyotype, have an increased risk for developing a type II germ cell tumor/cancer (GCC) [2]. GCCs arise from primordial germ cells (PGC) or gonocytes and can be subdivided into seminomas/dysgerminomas and non-seminomas with carcinoma *in situ* (CIS) or gonadoblastoma (GB) as precursor lesions [3]–[4]. GCC risk varies, but is estimated to be over 30% in patients with complete gonadal dysgenesis and is often bilateral [2].

Frasier syndrome (FS), currently classified as 46,XY DSD, complete gonadal dysgenesis, is characterized by gonadal dysgenesis, a high risk for development of a GCC and chronic renal failure in early adulthood. Usually patients with complete gonadal dysgenesis are not diagnosed at birth because of their normal female appearance of external genitalia. However, these patients will not develop secondary sex characteristics at pubertal age, and will generally attend the clinic because of primary amenorrhea, with hormonal analysis showing hypergonadotropic hypogonadism because of lack of gonadal function.

Wilm’s Tumor 1 (WT1) is an important regulator of early gonadal and kidney development [5]. It is expressed earlier in time than SRY in the urogenital ridge, from which the gonads and kidneys are derived. All known WT1 isoforms share four C-terminal zinc fingers which are necessary for DNA/RNA binding. The two major WT1 isoforms are produced by alternative splicing, resulting in an insertion (+KTS) or exclusion (-KTS) of lysine, threonine and serine between zinc fingers three and four. The –KTS isoform mainly plays a role in transcription, and *AMH* transcriptional activation in Sertoli cells [6]. The +KTS isoform is involved in RNA processing, and in the mouse plays a role in *Sry* regulation *in vivo* [7].

Essential in the process of sex determination is the presence of the sex determining region on the Y chromosome (SRY) gene [8], [9], [10]. *SRY* mutations residing in the HMG (High Mobility Group) domains are found in 10–15% of the 46,XY gonadal dysgenesis cases and affect binding to and bending of DNA or nuclear transport [11]–[14]. As a consequence these mutations can lead to an early error in the process of sex determination preventing proper formation of a testis. Specific intron 9 splice site mutations in *WT1* resulting in a decreased WT1+KTS isoform are typically found in FS patients, leading to a diminished expression of SRY and subsequently SOX9, thereby disturbing testicular development [15]. Furthermore, knockout mice for the +KTS isoform showed sex reversal in males [16]. Thus both *SRY* and *WT1* mutations can cause (complete) sex reversal.

A highly informative marker for the presence of type II GCCs (i.e. GB, CIS and their invasive counterparts dysgerminoma and seminoma as well as embryonal carcinoma) is the transcription factor OCT3/4, also known as POU5F1 [17]. OCT3/4 is involved in the regulation of pluripotency, is expressed in PGCs and gonocytes during normal gonadal development, is required for PGC survival, and is lost after maturation to pre-spermatogonia in males and oogonia in females [17]–[20]. In DSD patients OCT3/4 positivity of the germ cells might be due to maturation delay and not due to malignant transformation. To distinguish between these, Stem Cell Factor (SCF, also known as KITLG) has been shown to be informative [21]. GB arises in the context of granulosa cells, staining positive for FOXL2 and negative for SOX9 (a Sertoli cell marker), this in contrast to the precursor lesion arising in a testicular environment, being CIS, in which the supportive (Sertoli) cells are negative for FOXL2 and stain positive for SOX9 [22].

Here, we present a unique case with bilateral GB and dysgerminoma in an adult woman presenting with primary amenorrhea at the age of 22 years, who was initially diagnosed with 46,XY gonadal dysgenesis. Mutation analysis identified a novel missense mutation (c.383A>G, p.Lys128Arg) in the HMG domain of the *SRY*, which did not have a significant effect on transcriptional activation and nuclear import *in vitro*. Laparoscopy revealed streak ovaries with GB and dysgerminoma on both sides. During follow-up the patient developed progressive renal failure based on focal glomerulosclerosis. Subsequent analysis of the *WT1* gene revealed a splice site exon 9 mutation (IVS9+5 G>A) resulting in the final diagnosis FS. Sequence analysis of DNA from five additional FS patients with a proven *WT1* mutation for *SRY* mutations did not reveal any variants, indicating that the presence of mutations in both genes in FS patients is rare. To our knowledge this is the first case describing a patient with a mutation in both *WT1* and *SRY*, and underlines the importance of proper diagnosis, especially in patients with an increased risk for GCC, allowing early diagnosis and treatment, thus preventing the development of invasive cancer.

## Materials and Methods

### Tissue Samples

Anonymized tissue samples were collected from our diagnostic archives and diagnosed according to WHO standards [23] by an experienced pathologist in gonadal pathology, including GCC (JWO). Use of tissue samples for scientific reasons was approved by the Medical Ethical Committee ErasmusMC (MEC 02.981 and CCR2041). Samples were used according to the “Code for Proper Secondary Use of Human Tissue in The Netherlands” as developed by the Dutch Federation of Medical Scientific Societies (FMWV (Version 2002, updated 2011).

### Immunohistochemical Staining

Immunohistochemical staining was performed on formalin fixed paraffin embedded samples of 3 µm thickness. The antibodies directed against OCT3/4, c-KIT (CD117), Stem Cell Factor (SCF), Testis Specific Protein on the Y chromosome (TSPY), SOX9 and FOXL2 have been described before [21]–[22]. Briefly, after deparaffinization and 5 min incubation in 3% H_2_O_2_ for inactivating endogenous peroxidase activity, antigen retrieval was carried out by heating under pressure of up to 0.9 bar in an appropriate buffer. After blocking endogenous biotin using the Avidin/Biotin Blocking Kit (SP-2001; Vector Laboratories, Burlingame, CA, USA), sections were incubated either overnight at 4°C (SCF, TSPY) or for 2 h at room temperature (OCT3/4, SOX9, FOXL2) and detected using the appropriate biotinylated secondary antibodies and visualized using the avidin–biotin detection and substrate kits (Vector Laboratories).

### SRY Sequencing

Direct sequencing of the *SRY* gene on peripheral blood DNA from the patient was performed at the department of clinical genetics (reference sequence: NM_003140.1). For the additional samples DNA was isolated from either peripheral blood lymphocytes (4 patients) or from formalin fixed paraffin embedded material (from two independent blocks, 1 patient) according to standard procedures. SRY was PCR amplified, analyzed on a 1% agarose gel, purified using the Agencourt AMPure XP kit (Beckman Coulter genomics, Danvers, MA, USA) and Sanger sequencing was done according to standard procedures.

### SRY Transactivation Assay

DNA encoding wild type SRY, mutant SRY and SF1 were cloned into the pcDNA3 mammalian expression plasmid (Clontech, Mountain View, CA, USA), and sequence verified. To test for SRY activation of TESCO, *in vitro* luciferase assays were performed on a human embryonic kidney carcinoma cell line (HEK293T, ATCC, CRL-11268). Cells were cultured in DMEM, High Glucose, GlutaMAX media (Invitrogen, Life Technologies, Paisley, UK) containing 10% Fetal Bovine Serum, 1% sodium pyruvate and 1% penicillin-streptomycin. Cultures were grown at 37°C with 5% CO2. Cells were seeded in serum-free media 24 hours prior to transfection in 96-well tissue culture plates at a density of 30,000 cells per well.

Cells in each well were co-transfected with the reporter constructs TESCO-E1b-*Luc* (10 ng) or the empty vector E1b-*Luc* (8 ng), together with 40 ng of each of the expression constructs pcDNA3-SF1 and either pcDNA3-hSRY (wild-type) or pcDNA3-SRY-K128R (mutant). The reporter constructs contained the minimal E1b promoter driving a luciferase gene. pRL-TK-Renilla (Promega, Madison, WI, USA; 1 ng) was added to each well as an internal control. pcDNA3 and pUC DNA were added to make up a total of 100 ng DNA per well, and transfection was performed with 0.38 µl of FuGENE6 Transfection Reagent (Roche, Basel, Switzerland) following manufacturer’s instructions. Cells were lysed 48 h after transfection and firefly and Renilla luciferase activities were measured using the Dual-Luciferase Reporter Assay System (Promega).

Six independent assays were performed, each in triplicate. Firefly luciferase activity (Luc) was normalized against that of Renilla luciferase (Ren). Luc/Ren readings for TESCO-E1b-*Luc* were further normalized against that of E1b-*Luc* to obtain the fold change of TESCO activity over that of the empty vector. Fold change of the mutant SRY-K128R construct was then normalized against that of wild-type SRY. Data are therefore represented in the form of mean percentage of wild-type SRY fold change. Statistical analysis was performed by conducting an unpaired t-test.

### SRY Nuclear Import Assay

pcDNA3-FLAG-SRY plasmid has been described previously [13]. pcDNA3-FLAG-K128R was created using site-directed mutagenesis. All constructs were verified by sequencing.

HEK293T cells seeded in 6-well plates were transfected with 2 µg/well of either pcDNA3-FLAG-SRY wild-type or pcDNA3-FLAG-K128R mutant using Fugene 6 (Roche). After transfection, immunohistochemistry was carried out using mouse monoclonal antibody against FLAG tag (1∶400). The secondary antibody used was Alexa 488-conjugated donkey antimouse IgG (1∶500, Molecular Probes, Life technologies). DNA was stained with 0.1 µg/ml of 4',6-diamidino-2-phenylindole (Molecular Probes, Life technologies). Image analysis was performed by using NIH ImageJ (public domain software). Briefly, measurements were taken of the density of fluorescence from the cytoplasm and the nucleus with the background fluorescence subtracted from the equation: Fn/c = (n – bkgdn)/(cp – bkgdcp), where n = nucleus and bkgdn = background in the nucleus, cp = cytoplasm and bkgdcp = background in the cytoplasm.

### WT1 Mutation Analysis

Mutation analysis was performed at the department of clinical genetics of the Amsterdam Medical Center. Briefly: Exon 9 of the WT1 gene (NM_024426, but with the translation initiation codon starting at c.395), including flanking intronic sequences, was amplified by PCR followed by direct sequencing using Bigdye v1.1 chemistry and an ABI3100 sequencer (Life Technologies, Carlsbad, CA, USA). Sequences were analyzed using Codoncode Aligner (CodonCode Corporation, Dedham, MA, USA).

## Results

### Patient Clinical History

A phenotypically normal female presented at the outpatient clinic with primary amenorrhoea at the age of 22. She reported to have had some vaginal bleeding at the age of 13 and 14 years which she thought was the start of menarche. This together with the fact that she grew up in different families was the reason of her late clinical presentation. Patient history mentioned migraine and severe asthma for which she was treated with corticosteroids. Physical examination showed normal female external genitalia, with Tanner stage III breast development and stage II pubic hair development. She had a scoliosis and 2.5 cm difference in length of her legs. Hormonal analyses at the age of 22 and 23 years revealed low oestradiol: 11 and <10 pmol/L (normal 100–1000 pmol/L), testosterone: 1 nmol/L (normal 0.5–3 nmol/L), high FSH: 215 and 219 IU/L (normal 1–8 IU/L), and high LH: 78 and 75 IU/L (normal 2–8 IU/L) levels, indicating hypergonadotropic hypogonadism. Furthermore an increase in serum creatinine levels 111–217 umol/L (normal 90 umol/L) was found over the course of ten months suggestive of impaired kidney function, although not diagnosed at the time of presentation. Chromosome analysis on peripheral blood lymphocytes showed the presence of a 46,XY karyotype, and mutational analysis of the *SRY* gene revealed an, at that time, unclassified variant K128R (c.383 A>G, p.Lys128Arg). Based on these results the patient was diagnosed with 46,XY gonadal dysgenesis. Laparoscopic examination showed streak ovaries and a normal, but small, uterus. Because of the known tumor risk in these patients, both ovaries were removed during this intervention (for histology, see below).

Two months after gonadectomy the patient visited the emergency room with complaints of agonizing headache, which were caused by severe hypertension; her blood pressure was 200/127 mmHg, with a good response to treatment with Amlodipine. In addition, blood analyses showed severe renal failure and additional examinations showed that the progressive renal failure was due to primary focal glomerulosclerosis. The rapid progression of kidney failure together with the diagnosis of 46XY gonadal dysgenesis and bilateral GB and dysgerminoma (for histology, see below) triggered investigation for a *WT1* mutation. The patient is currently on haemodialysis and awaits kidney transplantation, which has to be postponed for five years (until 2014) due to the treatment of the GCC.

### Histological and Immunohistochemical Analysis

**Figure 1:**
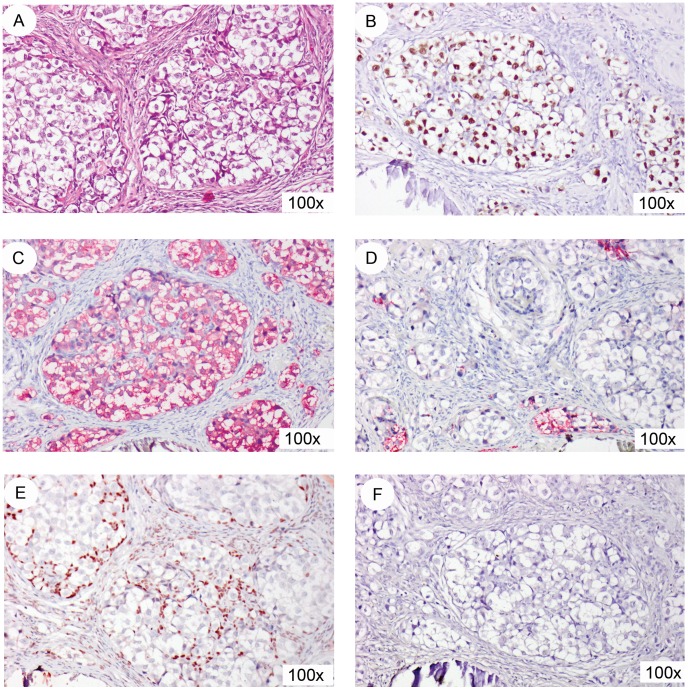
Immunohistochemical staining of the left GB lesion. (A) representative hematoxylin and eosin (HE) staining. The germ cells present in the GB stain positive for OCT3/4 (B, brown staining), TSPY (C, red staining), and SCF (D, red staining). Supportive cells in the GB stain positive for FOXL2 (E, brown staining), while SOX9 (F) is negative. All slides are counterstained with hematoxylin. Magnification 100x for all.

Histological examination of both gonads showed that GB and dysgerminoma was present in a dysgenetic histological context. The lesions on both sides were restricted to the gonad. A representative image of the hematoxylin and eosin (H&E) staining is shown in Figure 1A. In agreement with this diagnosis, the germ cells showed positive staining (shown only for the left GB lesion) for OCT3/4 (Figure 1B), TSPY (Figure 1C) and SCF (Figure 1D). In addition the supportive cells stained positive for FOXL2 (granulosa cell marker, Figure 1E) and were negative for SOX9 (Sertoli cell marker, Figure 1F). The GB removed from the other side showed a similar staining pattern for all markers investigated (data not shown). Both gonads showed multiple micro-calcifications (microlithiasis), represented in the images of Figure 1.

### Mutation Analysis and Functional Analysis of SRY

**Figure 2:**
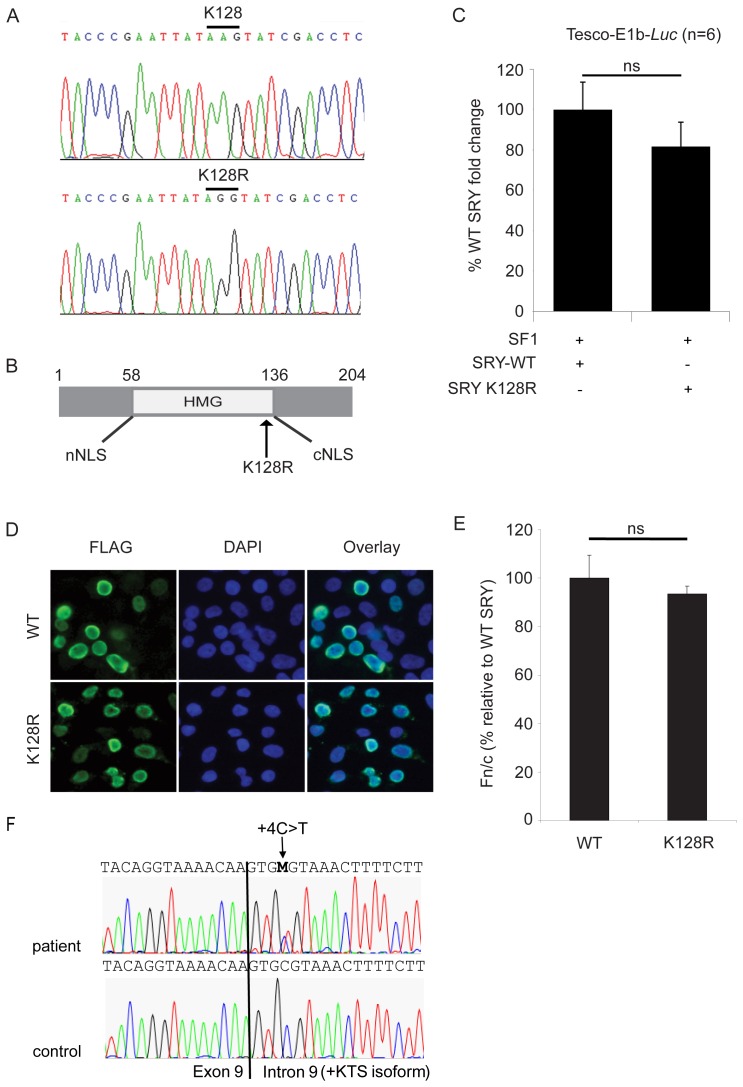
Mutational analysis of *SRY* and *WT1*. (A) wild type (upper panel, control) and mutated sequence (lower panel, patient) of *SRY*. (B) schematic representation of the SRY protein. The K128R mutation resides in the HMG domain, just before the cNLS. (C) *In vitro* luciferase assays of SRY-WT (wild-type) and SRY-K128R (mutant) in HEK293T cell line. Cells were co-transfected with TESCO-E1b-*Luc*, SF1 and WT or mutant SRY to assess for activation of TESCO. The mean percentages of fold change of luciferase activity of TESCO-E1b-*luc* over the empty vector, relative to WT SRY levels from six independent assays (each performed in triplicate) are shown. Error bars represent standard error of the mean (SEM). (D) pcDNA3-FLAG-SRY wild-type (WT, 2 µg) or pcDNA3-FLAG-SRY mutant (K128R, 2 µg) were transiently transfected into HEK293T cells using Fugene 6. Exogenous SRY (WT or K128R) expression was detected using a FLAG antibody and a green fluorescent Alexa-488 dye coupled secondary antibody. Nuclei were stained with 4′,6-diamino-2-phenylindole (DAPI). Both wild type and mutant SRY show strong nuclear staining. (E) SRY fluorescence was quantified as previously described [35]. Nuclear accumulation of SRY (WT or K128R) expressed as fluorescence in the nucleus over that in the cytoplasm (Fn/c) were background fluorescence has been subtracted. Measurements represent the average of 3 independent transfections. Results are relative to WT transfected cells (Fn/c given value of 100%). The number of cells analysed is n = 111 (WT) and n = 121 (K128R). Error bars represent the standard error of mean values. Two-tail t-Test of unpaired sample means was performed between WT transfected cells and mutant transfected cells and showed no significant differences. P = 0.49. (F) mutated sequence (upper panel, patient) and wild type sequence (lower panel, control) of *WT1*, showing the heterozygous +4C>T change.

Direct sequencing of the *SRY* gene showed the presence of a single nucleotide change at position 383 (A to G, see Figure 2A), resulting in a missense substitution (Lysine (K) to Arginine (R) amino acid change) at position 128 in the SRY protein (hemizygous pattern). A K128R missense mutation in SRY has not been reported to date. The K128R sequence variant is located within the HMG domain of SRY next to the C-terminal nuclear import signal (cNLS) (Figure 2B).

SRY activates *SOX9* expression together with SF1 via a testis-specific enhancer called TESCO, which is located approximately 13 kb upstream of *SOX9* [24]. The ability of the SRY K128R mutant form of SRY to activate *SOX9* via TESCO was analyzed. Results show that the K128R mutation of *SRY* did not significantly affect TESCO activity *in vitro* compared to wild-type SRY (Figure 2C), although a reduction of about 20% was observed.

As the K128R substitution is located next to the cNLS, the effect on nuclear import was also investigated using expression plasmids encoding wild-type and mutants full-length SRY transfected in HEK293T cells. The subcellular localization of SRY was determined 48 h after transfection using indirect immunofluorescence and quantified using image analysis (Figure 2D and 2E). Wild type SRY efficiently accumulated in the nucleus. The mutant K128R also showed a slight reduced but non-significant difference in nuclear accumulation compared to the wild type protein, indicating that the K128R mutation does not affect the nuclear import function of SRY.

### Mutation Analysis WT1 and Additional FS Samples Analyzed

As the patient had 46,XY gonadal dysgenesis together with renal failure (focal segmental glomerulosclerosis), and GB with dysgerminoma, without Wilm’s tumor, all pointing to FS, the *WT1* gene was analyzed. Direct sequencing of the *WT1* gene showed a single nucleotide change at the start of intron 9 at the position +4 (IVS9+4C > T) in a heterozygous state (Figure 2F), characteristic for FS.

To determine if *SRY* mutations together with *WT1* mutations were present in other DSD cases with the same clinical characteristics a review of the literature was done (Tables S1 and S2), showing that this has not been investigated to date. Therefore an additional five DNA samples from FS patients with a proven *WT1* mutation were analyzed for *SRY*, showing no aberrations in *SRY* in addition to the *WT1* mutation.

## Discussion

Sex determination and specifically testis differentiation in males is critically dependent on transcriptional regulation of a selective number of genes including *WT1, SRY*, and *SOX9* 25–26. Expression of the Y-chromosome located *SRY*, above a threshold and in a critical time window, is crucial in triggering testis formation. SRY will upregulate SOX9 which will orchestrate the formation of the pre-Sertoli cells and further regulates testis development. *WT1* is expressed in the gonadal ridges before the onset of SRY, and plays an important role in testicular as well as kidney formation. It has been suggested that the WT1+KTS isoform functions in terminal Sertoli cell differentiation and homeostasis through the maintenance of a critical level of *SRY* and *SOX9* expression [15].

*SRY* mutations play a role in 46,XY sex reversal (46,XY DSD) and in about 15% of 46,XY gonadal dysgenesis cases mutations are found [27]. The majority of mutations reside in the HMG domain, which is involved in the binding and bending of DNA. Besides these, mutations located in one of the NLSs have been reported, resulting in a reduced nuclear import of SRY. The K128R mutation described here does not lead to a statistically significant reduction in transactivational activity as ascertained by an *in-vitro* assay, although a minor reduction (about 20%) was observed. In addition, although located adjacent to the cNLS of SRY, the mutation does not result in a significant reduction in nuclear import of the protein. This suggests that the phenotype of the patient is not due to a nuclear import defect as has been observed in other cases [13]–[14], [28]. Although the lysine on position 128 is conserved between man and mouse, mutation of lysine on position 128 to arginine does not affect regulation of SRY subcellular distribution by (de-)acetylation via p300 [29]. Taken together, the results show that the mutation has little effect on the *in vitro* transactivation and nuclear import assays available. Therefore it is unlikely that the SRY K128R mutation has a significant effect on the (gonadal) phenotype of the patient has, although a more dramatic effect of the mutation in an *in vivo* situation cannot be ruled out.

Reviewing the literature shows that almost all gonadal dysgenesis cases with a proven *SRY* mutation (86 cases in total, Table S1) show a female phenotype (n = 81, 94%). Only a few cases show ambiguous genitalia (n = 4), and one patient has a male phenotype with ambiguous genitalia (respectively 5% and 1%). In a total of 61 cases gonadal histology was analyzed: 18 showed a GB (30%), one a dysgerminoma (1%) and two GB along with dysgerminoma (3%). This strongly shows the known increased GCC risk in these patients (34% in this cohort). Only a limited number of papers describe the functionality of *SRY* mutations (20 in total, 23%), and the effects range from completely abolished DNA binding to no differences in DNA binding when compared to wild type SRY. Based on these data, no genotype-phenotype correlation can be gathered (Table S1). In some cases the mutations described are also present (in mosaic form) in male family members, with one showing hypospadias and cryptorchidism, one diagnosed with a testicular seminoma, and one without GCC and a normal male phenotype [30]–[32] (refs 14, 15 and 54 in Table S1). Whether this is also the case in the patient described here, or the mutation arose *de novo,* cannot be investigated because family members are not available for analysis (see above).

The patient described here was initially diagnosed as a 46,XY DSD complete gonadal dysgenesis and a (until now unclassified) mutation in *SRY* was found (i.e. Swyer syndrome), associated with GB and dysgerminoma. However, upon follow-up the diagnosis of progressive renal failure based on focal segmental glomerulosclerosis, prompted analysis of the *WT1* gene. Initially the mild renal impairment found at presentation was not considered to be indicative to screen *WT1* for mutations.

Mutations in *WT1* play a role in 46,XY DSD (i.e. FS, Denys-Drash syndrome, and WAGR-syndrome), and those found in FS consist of *WT1* intron 9 splice-site mutations. These patients have complete 46,XY sex reversal, late onset kidney failure (between 10–20 years), focal segmental glomerulosclerosis, streak gonads, and a high risk for GB, but not Wilm’s tumors [33]. Sequence analysis of the *WT1* gene in the patient described here revealed a classic FS mutation in the intron 9 splice-site (IVS9+4 C>T). This ultimately results in the decrease of the +KTS isoform and it is known that the subsequent reversion in +KTS/−KTS ratio causes defects in the development of glomerular podocytes and male sex-determination, ultimately leading to _ephritic syndrome and male-to-female sex reversal, respectively [33]–[34]. Careful review of the literature revealed that this is the first patient described having both a *WT1* as well as a *SRY* mutation; however in almost all cases described a mutation screen of both *SRY* and *WT1* was not performed. Analysis of five additional FS patient samples with a proven *WT1* mutation by conventional Sanger sequencing of the *SRY* gene did not reveal any mutations. The majority of FS patients described in literature are phenotypically females (n = 48, 96%) and only two phenotypically males are presented (4%, Table S2). It also underlines the high incidence of GB and/or dysgerminoma in this patient group; 18 out of 39 patients with described gonadal histology showed GB (46%), in one patient carcinoma *in-situ* (CIS) is described, the precursor lesion of GCC in the testis, and in one patient GB next to CIS is described. Five patients had an invasive dysgerminoma next to the GB, one patient is described as having GB and a metastatic tumor, and one patient is mentioned as having dysgerminoma. In the other patients with described gonadal histology, the majority show streak gonads (n = 17, 44%), in one it is described as a dysgenetic gonad and in one no gonadal tissue could be found. For the other patients no gonadal histology was analyzed (n = 11).

It has been described that *SRY* and *SOX9* expression can be diminished in FS [15] and one could speculate that in the case presented here the effects from reduced *SRY* expression by a mutated *WT1* were exacerbated by the presence of the SRY K128R mutation, although a reduced SRY function could not be shown conclusively *in vitro*. This situation may have contributed to the maldevelopment of the gonads, thereby creating the micro-environment in which embryonic germ cells can survive, and are prone to become malignant. However, screening an additional five FS patients with a proven *WT1* mutation did not reveal any sequence variants in *SRY*. Although this is a limited series of these unique cases, it indicates that presence of *SRY* mutations in FS is rare.

To our knowledge this is the first patient described with a mutation in *SRY* together with a classical FS *WT1* mutation, and thus seems to be a rare condition. Nonetheless, in this patient an optimal diagnosis could have been made, if a screening for *WT1* mutation was performed at an earlier time point. The patient is currently on haemodialysis and awaits kidney transplantation, which has to be postponed for five years (until 2014) due to the GCC in her history. This case clearly demonstrates the significant role of proper diagnosis of the variants of DSD, especially in those with an increased risk for GCC, allowing early diagnosis and treatment, thus preventing the development of invasive cancer. The presence and type of *WT1* mutation has major consequences for the patient. We therefore suggest that *WT1* mutation screening should be performed in all patients with 46,XY gonadal dysgenesis, especially in case of an unclassified SRY variant, and not vice versa. In addition, careful evaluation of kidney function at early stage is recommended in these patients.

## Supporting Information

Table S1 ***SRY*** **mutation literature overview.** NA.: not available, GB: gonadoblastoma, DG: dysgerminoma, SE: seminoma, YST: yolk sac tumor, OT: ovotestis, S: streak, DS: dysgenetic testis, T: testis, ov st: ovarian stroma, GD: gonadal dysgenesis. POF: Premature Ovarian Failure.
(XLS)

Table S2 ***WT1*** **mutation literature overview.** GB: gonadoblastoma, CIS: carcinoma in situ, DG: dysgerminoma, d.g.: dysgenetic gonad, S: streak, NGT: no gonadal tissue, NA.: not available, GD: gonadal dysgenesis, FSGS: focal segmental glomerulosclerosis, LC: leydig cell.
(XLS)
